# Repurposing lipid-lowering drugs as potential treatment for acne vulgaris: a Mendelian randomization study

**DOI:** 10.3389/fmed.2024.1385948

**Published:** 2024-06-06

**Authors:** Man Fang, Jing Lei, Yue Zhang, Bo Zhang

**Affiliations:** ^1^Department of Plastic and Cosmetic Surgery, Hunan Provincial People's Hospital, The First Affiliated Hospital of Hunan Normal University, Changsha, China; ^2^College of Computer, Chengdu University, Chengdu, Sichuan, China; ^3^Xiangya Hospital, Central South University, Changsha, China

**Keywords:** acne vulgaris, lipid-lowering drugs, Mendelian randomization, GWAS data, lipid metabolism

## Abstract

**Background:**

Acne vulgaris, a chronic inflammatory skin condition predominantly seen in teenagers, impacts more than 640 million people worldwide. The potential use of lipid-lowering medications as a treatment for acne vulgaris remains underexplored. This study seeks to investigate the impact of lipid-lowering therapies on the risk of developing acne vulgaris using two-sample Mendelian randomization (MR) analysis.

**Method:**

The two-sample MR method was employed for analysis, and information on lipid-lowering drugs was obtained from the DrugBank and ChEMBL databases. The summary data for blood low-density lipoprotein (LDL) and triglycerides were sourced from the Global Lipids Genetics Consortium, while genome-wide association studies (GWAS) summary data for acne vulgaris were obtained from the FinnGen database. Heterogeneity was examined using the Q-test, horizontal pleiotropy was assessed using MR-Presso, and the robustness of analysis results was evaluated using leave-one-out analysis.

**Results:**

The MR analysis provided robust evidence for an association between lowering LDL cholesterol through two drug targets and acne vulgaris, with PCSK9 showing an odds ratio (OR) of 1.782 (95%CI: 1.129–2.812, *p* = 0.013) and LDL receptor (LDLR) with an OR of 1.581 (95%CI: 1.071–2.334, *p* = 0.021). Similarly, targeting the lowering of triglycerides through lipoprotein lipase (LPL) was significantly associated with an increased risk of acne vulgaris, indicated by an OR of 1.607 (95%CI: 1.124–2.299, *p* = 0.009).

**Conclusion:**

The current MR study presented suggestive evidence of a positive association between drugs targeting three genes (PCSK9, LDLR, and LPL) to lower lipids and a reduced risk of acne vulgaris.

## Introduction

Acne vulgaris is a chronic inflammatory disease of the pilosebaceous unit, predominantly found on the face, chest, upper back, and upper arms (1). It is the most common skin condition among teenagers and young adults (2), affecting up to 85% of this population (3). Acne vulgaris can lead to significant scarring of the skin, thus associated with serious psychological effects, including depression and anxiety as indicated by a meta-analytic review (4, 5). Effective treatment of acne vulgaris has been shown to significantly improve quality of life (6). However, treating this condition remains a challenge. Current treatment options, such as regular skin care, topical or oral retinoids, antibiotics, benzoyl peroxide, or azelaic acid, often fall short in terms of efficacy and safety (3). Consequently, there is a pressing need for research into more effective treatments for acne vulgaris.

Acne vulgaris is influenced by four well-established pathological factors: (1) increased sebum production; (2) irregular follicular desquamation; (3) *Propionibacterium acnes* proliferation; and (4) inflammation of the affected area (7). Recent approaches to treatment have included therapies targeting *Propionibacterium acnes* (8). A study has shown that males with higher levels of total cholesterol, LDL, and triglycerides tend to have more severe acne vulgaris (9). Furthermore, disruptions in the sphingolipid metabolic pathway were observed in a metabolomics analysis of plasma from patients with moderate-to-severe acne (10). These findings indicate that lipid-lowering medications could potentially serve as preventive treatments for acne vulgaris.

In the Mendelian randomization (MR) approach, genetic variants are used as tools to determine whether a risk factor causally influences a health outcome (11). This method can help ascertain the plausibility of a correlation between an observation and its causal impact (12), while also minimizing the issues of confounding bias and reverse causality. Therefore, we employed a two-sample MR analysis in our study to explore the potential causal effect of lipid-lowering drugs (including statins, alirocumab, ezetimibe, mipomersen, evinacumab, fenofibrate, acipimox, and volanesorsen) on acne vulgaris.

## Methods

### Study design

**Figure 1:**
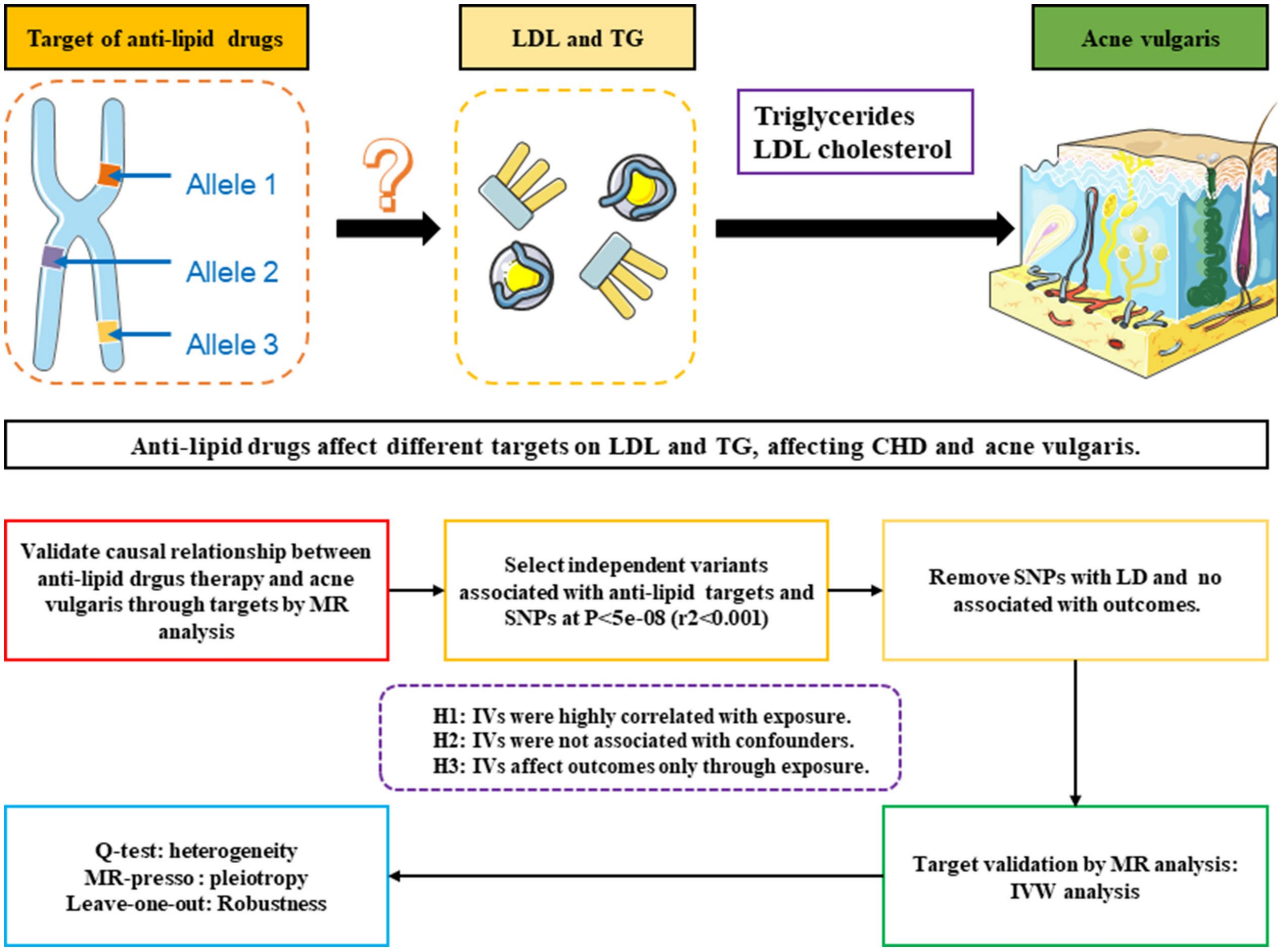
Schematic and flowchart progress of anti-lipid drugs therapy on different targets on LDL and TG, affecting CHD and acne vulgaris.

The two-sample MR method was utilized to examine the effects of current lipid-lowering therapies, including those targeting LDL and triglycerides, on the risk of acne vulgaris. Instrumental variables (IVs), specifically single nucleotide polymorphisms (SNPs) near the target genes of various lipid-lowering therapies, were derived from large-scale genome-wide association studies (GWAS) data representing exposure to these therapies (13). To ensure the method’s reliability, a positive control analysis was initially conducted to confirm the known treatment effect of lipid-lowering therapies on coronary heart diseases (CHD), given the established benefit of these therapies in reducing CHD risk. After confirming their effectiveness in the CHD context, these IVs were further employed to explore the causal relationship between lipid-lowering therapies and acne vulgaris. The overall methodology of this MR study is summarized in Figure 1.

### Target gene identification of lipid-lowering therapies

Lipid-lowering drugs can be categorized into two main types based on their effects on serum lipids: those that lower LDL cholesterol and those that lower triglycerides. First, we identified eight classes of lipid-lowering drugs from the Mayo Clinic website,^1^ which included statins, alirocumab, ezetimibe, mipomersen, evinacumab, fenofibrate, volanesorsen, and acipimox. Among these, statins, alirocumab, ezetimibe, mipomersen, and evinacumab are known for their LDL-lowering effects, while Evinacumab, fenofibrate, and volanesorsen target triglyceride levels. Furthermore, this study considers two critical genes involved in serum lipid metabolism: the LDL Receptor (LDLR) and Lipoprotein Lipase (LPL). Subsequently, we gathered information on target genes for these drugs from the DrugBank^2^ and ChEMBL^3^ databases. The target genes suitable for further analysis were identified from both databases. As illustrated in Table 1, eight target genes were initially identified as treatment targets for LDL-lowering drugs: HMG-CoA reductase (HMGCR), Proprotein convertase subtilisin/kexin type 9 (PCSK9), Niemann-Pick C1-like protein 1 (NPC1L1), mRNA of ApoB-100 (APOB), Angiopoietin-related protein 3 (ANGPTL3), Peroxisome proliferator-activated receptor alpha (PPARA), LDLR, and LPL. Furthermore, as shown in Table 2, five target genes were identified as treatment targets for triglyceride-lowering drugs: ANGPTL3, PPARA, APOC3, LDLR, and LPL.

**Table 1:** Target gene information of lipid-lowering drugs derived from different drug-gene interaction databases.

Drug class	Databases	Target protein	Encoding gene	Selected target
Statins	DrugBank	3-hydroxy-3-methylglutaryl-coenzyme A reductase Integrin alpha-L Histone deacetylase 2	HMGCR ITGAL HDAC2	HMGCR
	ChEMBL	HMG-CoA reductase	HMGCR	
Alirocumab	DrugBank	Proprotein convertase subtilisin/kexin type 9	PCSK9	PCSK9
	ChEMBL	Subtilisin/kexin type 9	PCSK9	
Ezetimibe	DrugBank	Niemann-Pick C1-like protein 1 Sterol O-acyltransferase 1 Aminopeptidase N	NPC1L1 SOAT1 ANPEP	NPC1L1
	ChEMBL	Niemann-Pick C1-like protein 1	NPC1L1	
Mipomersen	DrugBank	mRNA of ApoB-100	APOB	APOB
	ChEMBL	Apo-B 100 mRNA	APOB	
Evinacumab	DrugBank	Angiopoietin-related protein 3	ANGPTL3	ANGPTL3
	ChEMBL	Angiopoietin-related protein 3	ANGPTL3	
Fenofibrate	DrugBank	Peroxisome proliferator-activated receptor alpha	PPARA	PPARA
	ChEMBL	Peroxisome proliferator-activated receptor alpha	PPARA	
Acipimox	DrugBank	Non	Non	Non
	ChEMBL	Non	Non	
		LDL Receptor	LDLR	LDLR
		Lipoprotein Lipase	LPL	LPL

DrugBank: https://go.drugbank.com/; ChEMBL: https://www.ebi.ac.uk/chembl/.

**Table 2:** Target gene information of triglyceride-lowering drugs derived from different drug-gene interaction databases.

Drug class	Databases	Target protein	Encoding gene	Selected target
Evinacumab	DrugBank	Angiopoietin-related protein 3	ANGPTL3	ANGPTL3
	ChEMBL	Angiopoietin-related protein 3	ANGPTL3	
Fenofibrate	DrugBank	Peroxisome proliferator-activated receptor alpha	PPARA	PPARA
	ChEMBL	Peroxisome proliferator-activated receptor alpha	PPARA	
Volanesorsen	DrugBank	Apolipoprotein C-III	APOC3	APOC3
	ChEMBL	Apolipoprotein C-III mRNA 3’UTR	APOC3	
		LDL Receptor	LDLR	LDLR
		Lipoprotein Lipase	LPL	LPL

### GWAS summary data selection of exposures and outcomes

All the GWAS summary data for serum LDL cholesterol, triglycerides, and CHD used in this study were obtained from previous GWAS studies, with detailed information presented in Table 3. The largest GWAS summary dataset for serum LDL-cholesterol and triglycerides, which included 188,577 participants who had undergone serum lipid testing, was used as the exposure variable (14). In this dataset, blood lipid levels were measured after an 8 h fast, and the results were adjusted for age, sex, and normalized. For the positive control analysis, the largest GWAS summary dataset for CHD, which included 60,801 cases and 123,504 controls, was used (15). The largest dataset for acne vulgaris, comprising 1,092 cases and 211,139 controls, was sourced from the FinnGen database.^4^

**Table 3:** The baseline information of GWAS summary data related to LDL-cholesterol, triglyceride, coronary heart disease, and acne vulgaris.

Datasets	Source	Year	PMID	Population	Case (*n*)	Control (*n*)
LDL cholesterol	GLGC	2013	24,097,068	Mixed	188,577	
Triglycerides	GLGC	2013	24,097,068	Mixed	188,577	
Coronary heart disease	CARDIoGRAMplusC4D	2021	26,343,387	Mixed	60,801	123,504
Acne vulgaris	FinnGen	2021	NA	European	1,092	211,139

GAGC, Global Lipids Genetics Consortium (http://lipidgenetics.org/); CARDIoGRAMplusC4D, Coronary ARtery DIsease Genome wide Replication and Meta-analysis (CARDIoGRAM) plus The Coronary Artery Disease (C4D) Genetics (http://www.cardiogramplusc4d.org/).

### Mendelian randomization analysis

A two-sample MR analysis using the inverse-variance weighted (IVW) method was performed to estimate the causal effects of lipid-lowering drugs on acne vulgaris (16). First, IVs representing exposure to these drugs were identified. For an IV to be valid, it had to meet three criteria: (1) a strong correlation with the exposure, (2) no association with potential confounders, and (3) an influence on the outcome exclusively through the exposure. We utilized the IVW-MR method to gather information on genetic instrumental variables associated with different drug exposures. This analysis focused on eight target genes for LDL-lowering treatment and five target genes for triglyceride-lowering treatment. To identify IVs, SNPs significantly associated with LDL cholesterol or triglycerides (*p* < 5.0 × 10^−8) and located within a 100 kb window of each target gene were selected as potential genetic proxies. A clumping parameter for linkage disequilibrium (LD) was set at *r*^2^ < 0.001 to ensure the independence of SNPs (17). In addition, the F-statistic for each SNP and the mean F-statistic for all selected SNPs were calculated to assess their effectiveness as genetic proxies for different lipid-lowering treatments, with an *F*-value over 10 considered indicative of strong instrumental variables. Before conducting the MR analysis between lipid-lowering treatments and acne vulgaris, an MR analysis between lipid-lowering treatments and coronary heart disease (CHD) was performed to confirm the appropriateness of these genetic targets for representing the lipid-lowering therapies. Only targets that significantly impacted the risk of CHD were used for further MR analysis to explore the relationship between lipid-lowering therapies and acne vulgaris.

### Sensitivity analysis

Following the two-sample MR analysis, a sensitivity analysis was conducted to assess the stability of the results. The MR-PRESSO analysis was used to determine whether any SNPs exhibited horizontal pleiotropy with the outcome. A *p*-value of less than 0.05 in the global test of MR-PRESSO indicates significant horizontal pleiotropy, necessitating the removal of these SNPs. Furthermore, the MR-pleiotropy test was used to identify significant horizontal pleiotropy, with a *p*-value of less than 0.05 considered statistically significant. To assess heterogeneity in the MR analysis results, Cochrane’s Q-test was employed using MR-Egger and IVW methods, helping identify significant heterogeneity that could arise from population differences between the exposure and the outcome. Furthermore, the leave-one-out test was used to verify the robustness of the MR analysis results. In this test, SNPs were sequentially removed from the instrumental variable set to observe the impact of the remaining SNPs on the outcome.

All analyses were conducted using R software, alongside appropriate R packages.

## Results

### Genetic proxy for lipid-lowering therapies

Table 1, 2 shows that after searching the DrugBank and ChEMBL databases for drug-target interactions, 9 target genes were primarily identified for inclusion: HMGCR, PCSK9, NPC1L1, APOB, ANGPTL3, PPARA, APOC3, LDLR, and LPL. Due to their significant roles in regulating lipid metabolism, LDLR and LPL were also included as potential targets for lipid-lowering therapies.

We examined SNPs located within 100 kb of target gene regions to identify those that could serve as proxies for exposure to LDL-lowering or triglyceride-lowering drugs. Supplementary Table S1 shows seven SNPs within the HMGCR gene region significantly associated with LDL levels, suggesting their suitability as proxies for statin therapy. Supplementary Table S2 reveals twelve SNPs associated with LDL levels and located near the PCSK9 gene region, indicating potential proxies for alirocumab therapy. Supplementary Table S3 shows that three SNPs within the NPC1L1 gene region were significantly linked to LDL levels, serving as proxies for ezetimibe therapy.

For other LDL-lowering therapies, we identified suitable genetic proxies as follows: twenty SNPs within the APOB gene region for mipomersen therapy, three SNPs within the ANGPTL3 gene region for Evinacumab therapy, and one SNP within the PPARA gene region for fenofibrate therapy, as shown in Supplementary Tables S3–S6. In addition, 14 SNPs were identified as proxies for therapies targeting the LDLR gene (Supplementary Table S7).

Regarding triglyceride-lowering therapies, we identified proxies for evinacumab therapy with four SNPs within the ANGPTL3 gene region (Supplementary Table S8) and for volanesorsen therapy, four SNPs within the APOC3 gene region (Supplementary Table S9). In addition, 24 SNPs were identified as proxies for therapies targeting the LPL gene, indicative of potential triglyceride-lowering effects (Supplementary Table S10).

### Positive control analysis and sensitivity analysis

The MR analysis for the impact of various LDL-lowering therapies on CHD is detailed in Table 4. All IVs had a mean F-statistic value over 10, suggesting strong instrumental strength. The results showed that an increased LDL level associated with the APOB gene raises the risk of CHD (OR=1.243, 95%CI: 1.106–1.397). Similarly, an increase in LDL level linked to the HMGCR gene was associated with a higher CHD risk (OR=1.444, 95%CI: 1.240–1.682). Increased CHD risks were also observed with LDLR (OR=1.820, 95%CI: 1.571–2.108), NPC1L1 (OR=1.655, 95%CI: 1.201–2.281), and PCSK9 (OR=1.523, 95%CI: 1.303–1.779) target genes. The sensitivity analysis summarized in Supplementary Table S11 showed no significant heterogeneity for HMGCR, PCSK9, NPC1L1, ANGPTL3, and LDLR targets. However, potential heterogeneity was noted in the analysis involving the APOB gene (I^2^=43.432).

**Table 4:** The MR analysis of the effect of different lowering LDL cholesterol on coronary heart disease.

Targets	*F*	SNP (*n*)	Beta (95%CI)	Se	OR (95%CI)	*p*
ANGPTL3	133.2343	3	0.240 (−0.014, 0.495)	0.130	1.272 (0.986, 1.641)	0.064
APOB	220.249859	19	0.217 (0.101, 0.334)	0.060	1.243 (1.106, 1.397)	<0.001
HMGCR	151.0792208	7	0.368 (0.215, 0.520)	0.078	1.444 (1.240, 1.682)	2.18E-06
LDLR	117.1812478	10	0.599 (0.452, 0.746)	0.075	1.820 (1.571, 2.108)	1.38E-15
NPC1L1	94.5278694	3	0.504 (0.183, 0.825)	0.164	1.655 (1.201, 2.281)	0.002
PCSK9	130.3062592	10	0.421 (0.265, 0.576)	0.079	1.523 (1.303, 1.779)	1.18E-07
PPARA		0				
LPL		0				

The MR analysis of different triglyceride-lowering therapies on CHD, also summarized in Table 4, indicated that increased LDL levels driven by the APOC3 gene (OR=1.242, 95%CI: 1.115–1.384) and the LPL gene (OR=1.534, 95%CI: 1.399–1.681) would increase the risk of CHD. Conversely, no significant increase in risk was associated with the ANGPTL3 target (OR=1.272, 95%CI: 0.986–1.641). In the analysis of exposure to triglyceride-lowering therapies and CHD, the sensitivity analysis revealed no significant heterogeneity (see Table 5)

**Table 5:** The MR analysis of the effect of different triglyceride-lowering therapy on coronary heart disease.

Targets	*F*	SNP (*n*)	Beta (95%CI)	Se	OR (95%CI)	*p*
ANGPTL3	209.4852653	3	0.240 (−0.014, 0.495)	0.130	1.272 (0.986, 1.641)	0.064
APOC3	298.7056209	10	0.217 (0.108, 0.325)	0.055	1.242 (1.115, 1.384)	8.74E-05
LPL	200.0503049	22	0.428 (0.336, 0.519)	0.047	1.534 (1.399, 1.681)	6.56E-20
LDLR		0				
PPARA		0				

### Lipid-lowering therapy and acne vulgaris

Based on the MR analysis results from the positive control analysis, we included five targets from LDL-lowering therapies and two from triglyceride-lowering therapies to examine their association with acne vulgaris. As detailed in Table 6, the increase in blood LDL level driven by the PCSK9 gene, which represents exposure to alirocumab therapy, was positively linked to the risk of acne vulgaris (OR=1.782, 95%CI: 1.129, 2.812). Similarly, an increase in LDL level due to LDLR gene activation was significantly associated with an increased risk of acne vulgaris (OR=1.581, 95%CI: 1.072, 2.334). However, the other three LDL-lowering therapies, targeting HMGCR, NPC1L1, and APOB, showed no significant causal effect on acne vulgaris risk. The sensitivity analysis for the MR analysis between LDL-lowering therapies and acne vulgaris, summarized in Supplementary Table S13, revealed no significant heterogeneity except for the APOB target.

**Table 6:** Mendelian randomization analysis results of different LDL-cholesterol-lowering therapy on acne vulgaris using IVW methods.

Targets	*F*	SNPs	Beta (95%CI)	Se	OR (95%CI)	*P*
HMGCR		7	−0.200 (−0.896, 0.497)	0.355	0.819 (0.408, 1.644)	0.574
PCSK9		12	0.578 (0.122, 1.034)	0.233	1.782 (1.129, 2.812)	0.013
NPC1L1		3	−0.651 (−2.020, 0.718)	0.699	0.522 (0.133, 2.051)	0.352
APOB		20	−0.324 (−0.767, 0.118)	0.226	0.723 (0.464, 1.126)	0.151
LDLR		14	0.458 (0.069, 0.847)	0.199	1.581 (1.071, 2.334)	0.021

**Figure 2:**
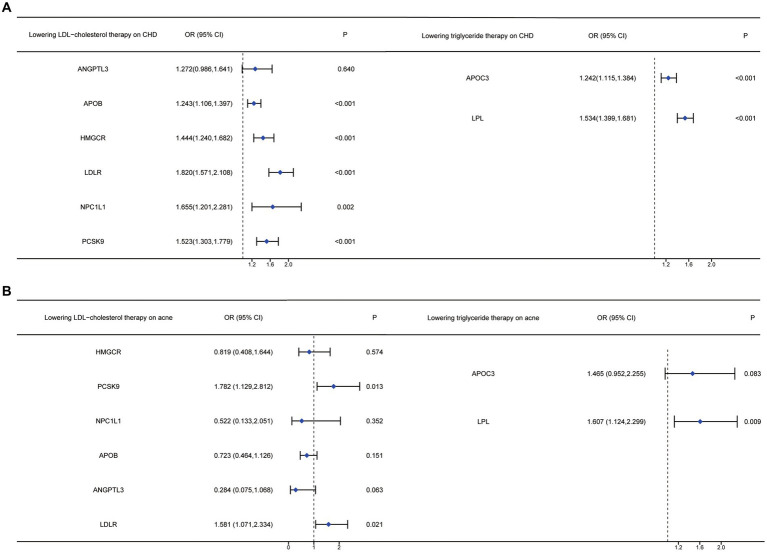
Forest plots of anti-lipid drugs therapy on different targets on LDL and TG, affecting CHD and acne vulgaris.

In Table 7, the MR analysis demonstrated that an increased triglyceride level driven by the LPL gene significantly affected the risk of acne vulgaris (OR=1.607, 95%CI: 1.124, 2.299). However, there was no significant association found between acne vulgaris and exposure to triglyceride-lowering therapy driven by the APOC3 gene (Figure 2). The sensitivity analysis showed no significant heterogeneity in the MR analysis between triglyceride-lowering therapy and acne vulgaris (Supplementary Table S14).

**Table 7:** Mendelian randomization analysis results of different triglyceride-lowering therapy on acne vulgaris using IVW methods.

Targets	*F*	SNPs	Beta (95%CI)	Se	OR (95%CI)	*p*-val
APOC3		10	0.382 (−0.049, 0.813)	0.220	1.465 (0.952, 2.255)	0.083
LPL		24	0.475 (0.117, 0.832)	0.182	1.607 (1.124, 2.299)	0.009

### Sensitivity analyses

The sensitivity analysis of the association between various lipid-lowering drugs (both triglyceride-lowering and LDL cholesterol-lowering) and CHD was conducted using MR-Egger and IVW methods, as detailed in Supplementary Tables S11, S12. The stability of the MR analysis results was confirmed through Cochrane’s Q-test, MR-Egger intercept tests, and MR-PRESSO, as indicated in Supplementary Figures S1–9A,B. Furthermore, the leave-one-out analysis and funnel plots further demonstrated the robustness of the MR analysis results (Supplementary Figures S1–9C,D).

## Discussion

Our research utilized a two-sample MR analysis to explore the potential effects of lipid-lowering drugs on acne vulgaris. This MR study offered preliminary evidence of a beneficial association between drugs such as alirocumab (proprotein convertase subtilisin/kexin type 9 (PCSK9) inhibitors), LDLR, and LPL inhibitors, and a reduced risk of acne vulgaris.

Acne vulgaris is a widespread inflammatory skin condition (18). Globally, it affects over 640 million people (19). In the United States, approximately 90% of teenagers experience acne, with nearly half continuing to experience symptoms into adulthood (20). Annually, nearly 3.5 million people in the United Kingdom are affected by acne vulgaris (21). In China, acne vulgaris shows higher prevalence rates among male subjects than female subjects, and among primary and secondary school students compared to college students (22). Acne can lead to permanent scarring and has significant negative psychosocial impacts (23). Studies link acne vulgaris with various mental health issues, including a higher incidence of mood disorders, psychiatric hospitalizations, school absences, unemployment, and suicidality (24). Effective treatment has been shown to improve quality of life (6). Furthermore, acne vulgaris imposes a considerable societal and healthcare burden (25). Given its high prevalence and serious consequences, it is crucial to identify effective and safe treatments for acne vulgaris.

According to a review of acne vulgaris treatments, primary treatments include topical retinoids, topical and oral antibiotics, benzoyl peroxide, hormone-based therapies, isotretinoin, and diet adjustment (24). However, issues such as side effects and unresponsiveness can affect adherence to topical therapies, diminishing treatment efficacy. The various drawbacks of current treatments highlight the need for developing new and effective therapies for persistent or relapsing acne vulgaris (26). Repurposing existing medications can be much more cost-effective and faster than developing new drugs (12).

Acne vulgaris is a complex condition characterized by abnormal follicular keratinization, increased sebum production, *Propionibacterium acnes* proliferation, and inflammation (27). A meta-analysis has demonstrated a significant association between several risk factors—such as family history, age, BMI, and skin type—and the presentation or severity of acne vulgaris (28). Previous research has identified acne vulgaris as one of the metabolic diseases driven by the mechanistic target of rapamycin complex 1 (mTORC1) (29). Reviews have discussed how lipid alterations and metabolic disturbances are characteristic of acne vulgaris (30). Notably, lipid alterations in acne include increased total lipid concentrations, fatty acids, sterol lipids, glycerophospholipids, unsaturated fatty acids, squalene, triglycerides, ceramides, and wax esters (31). Squalene may activate lipoxygenase, leading to the release of proinflammatory cytokines in acne (31). Furthermore, an imbalance in the ratio of saturated to unsaturated fatty acids in sebum can affect lipid metabolism and the inflammatory response (31). Research has shown that TREM2 macrophages, induced by human lipids, drive inflammation in acne lesions (32). Furthermore, lipid-lowering drugs are crucial in managing various skin conditions, and these drugs have been used to treat dermatomyositis (33). A review indicated that systemic lipid-lowering medications might lead to eczema, ichthyosis, or psoriasis as side effects, highlighting the role of lipid metabolism in these skin diseases (34). Statins and fibrates are also being investigated for their potential effects in preventing melanoma (35). This raises the question of whether lipid-lowering drugs could also benefit patients with acne vulgaris.

Until now, statins have been widely used in clinical treatments for lipid-associated diseases and are the most commonly prescribed class of lipid-lowering drugs known as HMG-CoA reductase (HMGCR) inhibitors (12). However, our results do not indicate any association between statins and acne vulgaris. Our study suggests that alirocumab, a PCSK9 inhibitor, may reduce the risk of acne vulgaris. PCSK9 significantly influences LDL-C metabolism through its role in LDL receptor degradation (36). A review indicates that PCSK9 inhibitors such as alirocumab can reduce plasma LDL-C levels by approximately 60% (37). Consequently, we hypothesize that acne vulgaris might be linked to LDLR degradation, positioning alirocumab as a potential therapeutic option. In addition, our findings suggest that both LPL and LDLR could lower the risk of acne vulgaris. LPL, a multifunctional enzyme critical in lipoprotein metabolism, is produced in various tissues including adipose tissue, cardiac and skeletal muscles, islets, and macrophages (38). It primarily converts lipoprotein triglycerides into free fatty acids and monoglycerides (39). The LDLR protein family is crucial in lipoprotein transport, with high LDL cholesterol levels being a common risk marker for coronary heart disease and other atherosclerosis-related conditions (40). Based on prior research and our findings, LPL and LDLR could be considered significant targets for acne vulgaris treatment.

Randomized clinical trials (RCTs) are the gold standard for determining the causal effects of drugs. However, RCTs can be expensive and impractical in some cases. Mendelian randomization (MR), which utilizes the natural random distribution of genetic variants, offers a way to explore the observational association between a drug and a disease while minimizing confounding bias and reverse causality (41). Consequently, we conducted a two-sample MR analysis to investigate the potential effects of lipid-lowering drugs on acne vulgaris.

Our study has several strengths. To the best of our knowledge, it is the first study to explore the impact of lipid-lowering drugs on acne vulgaris using MR. This method helps reduce confounding bias significantly. Furthermore, we used positive control analysis (coronary heart disease, CHD) and various MR techniques to enhance the validity of genetic instruments and the reliability of our findings. Despite these advantages, MR studies are not without limitations. Due to the inherent characteristics of MR, issues such as trait heterogeneity and canalization cannot be bypassed. Furthermore, our study did not perform reverse MR analysis, and the number of acne vulgaris cases was relatively small. Future studies with larger populations are needed to confirm our findings. In addition, it was necessary to perform experiments to detect the treatment effect of these lipid-lowering drugs on acne, and it would be necessary to detect the expression changes of these lipid metabolisms-related genes between acne patients and normal people. To summarize, we considered the effect of single drugs on acne vulgaris, and future studies should investigate the impact of the combination of various lipid-lowering drugs.

## Conclusion

Our results suggest that lipid-lowering drugs may reduce the risk of acne vulgaris. This study highlights the potential ancillary benefits of these medications on acne vulgaris beyond their primary function of reducing lipid levels, suggesting a possible therapeutic advantage for treating acne vulgaris. Further research into repurposing or retargeting these lipid-lowering drugs is essential to accelerate the development of effective treatments for acne vulgaris.

## Data availability statement

The original contributions presented in the study are included in the article/Supplementary materials, further inquiries can be directed to the corresponding author.

## Author contributions

MF: Data curation, Investigation, Software, Writing – original draft. JL: Data curation, Methodology, Software, Writing – original draft. YZ: Data curation, Formal analysis, Software, Writing – original draft. BZ: Conceptualization, Funding acquisition, Project administration, Resources, Supervision, Validation, Visualization, Writing – review & editing.

## Footnotes

^1^ https://www.mayoclinic.org/diseases-conditions/high-blood-cholesterol/in-depth/cholesterol-medications/art-20050958

^2^ https://go.drugbank.com/

^3^ https://www.ebi.ac.uk/chembl/

^4^ https://r8.finngen.fi/
