# Supplementary material for: Repurposing lipid-lowering drugs as potential treatment for acne vulgaris: a Mendelian randomization study
Source: Front Med (Lausanne). 2024 Jun 6;11:1385948. doi: 10.3389/fmed.2024.1385948 (PMC11187329; doi:10.3389/fmed.2024.1385948)
Supplement: Supplementary file 1 [file Data_Sheet_1.DOCX]

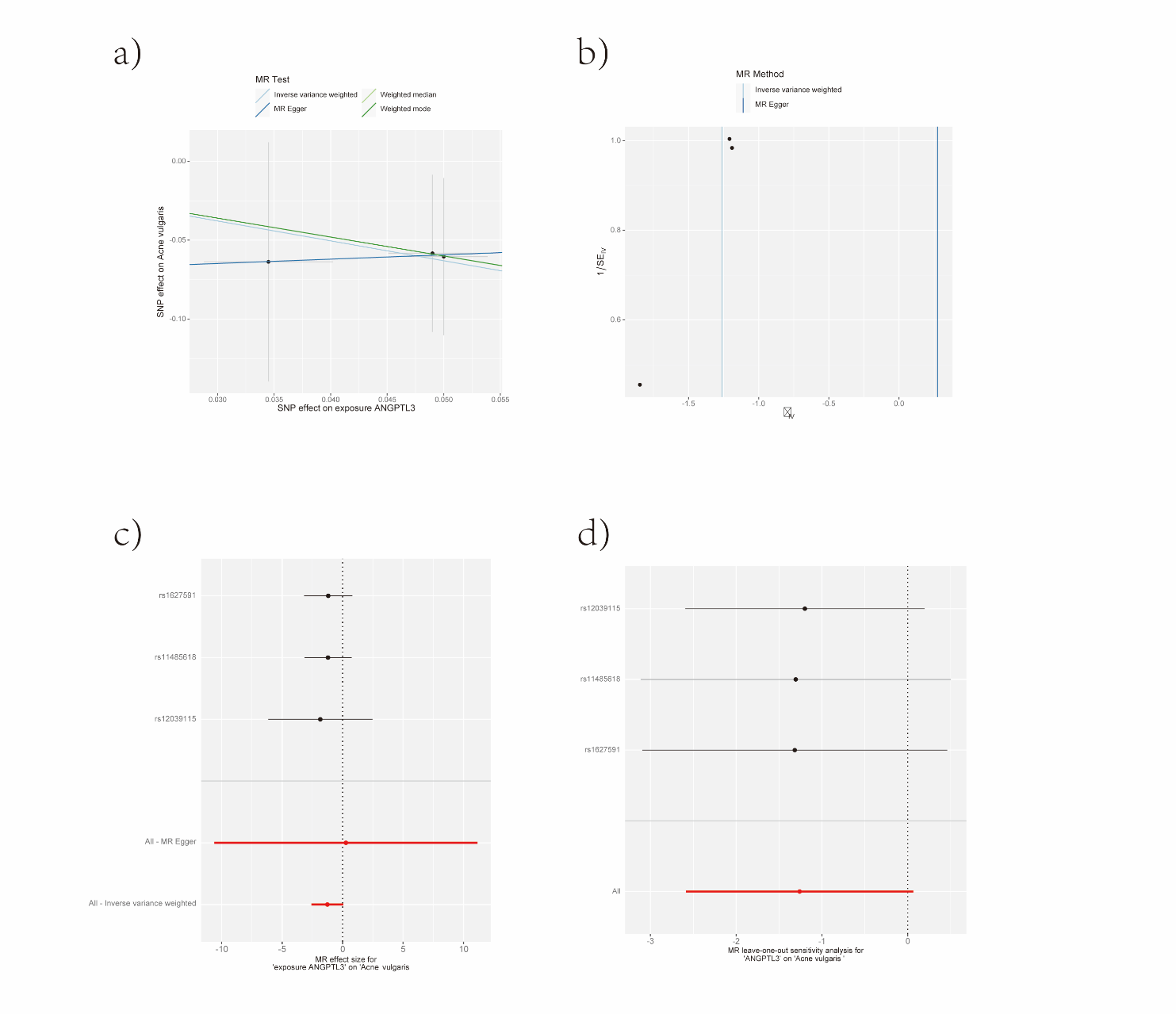


Figure S1. a) Scatter diagram b) funnel plot c) forest plot and d) leave-one-out test of treatment by LDL on acne vulgaris through gene ANGPTL3.


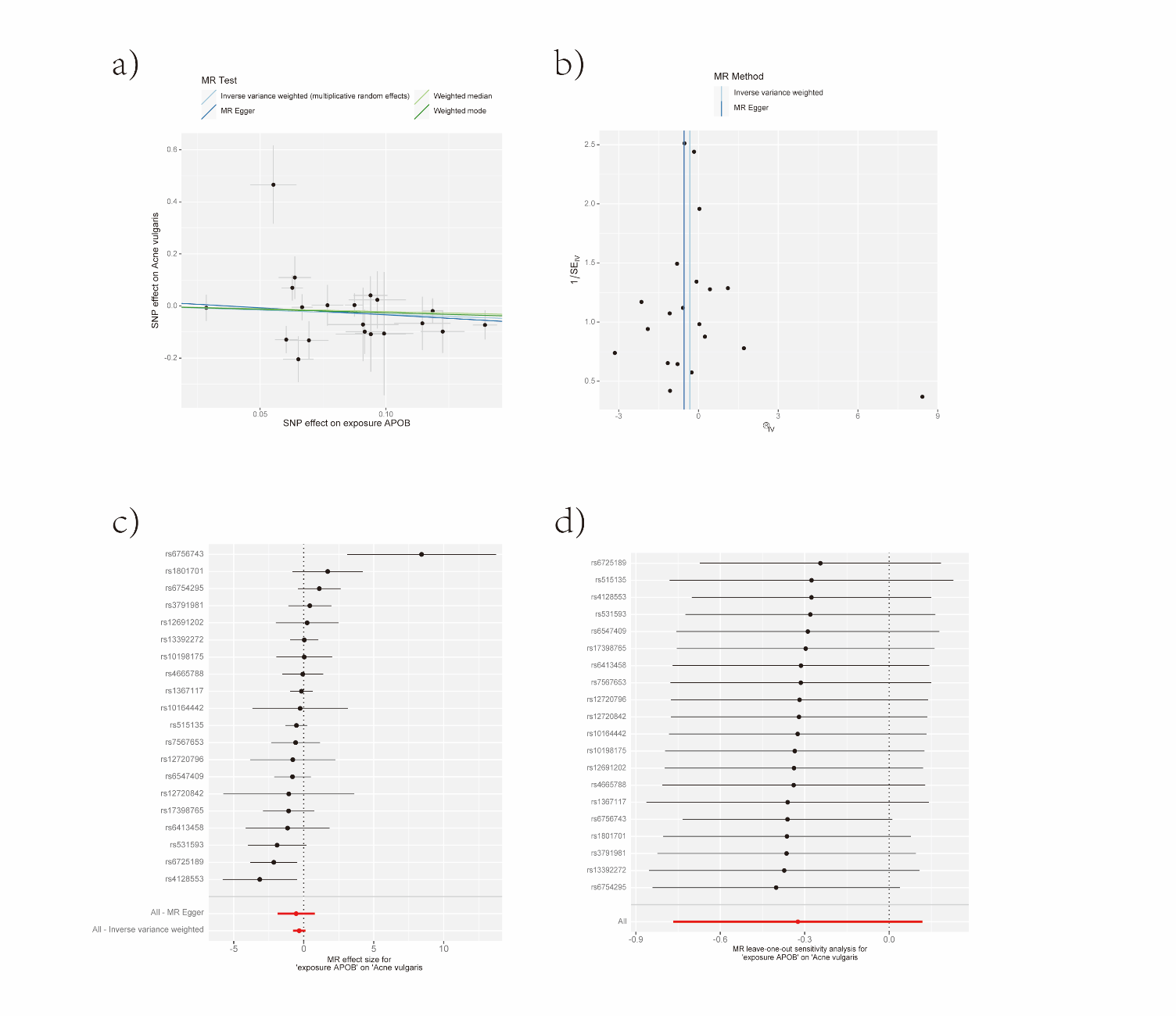


Figure S2. a) Scatter diagram b) funnel plot c) forest plot and d) leave-one-out test of treatment by LDL on acne vulgaris through gene APOB.


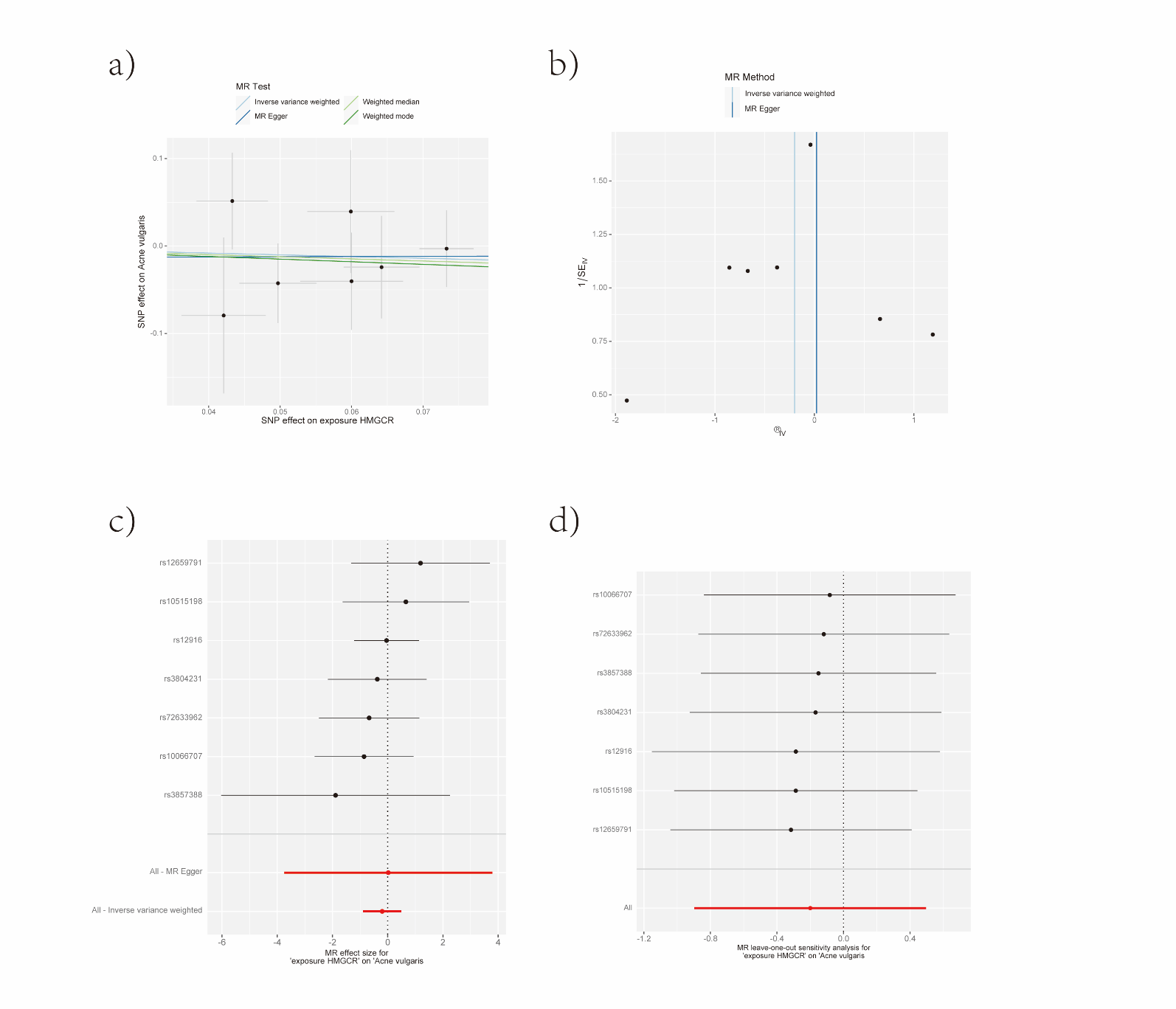


Figure S3. a) Scatter diagram b) funnel plot c) forest plot and d) leave-one-out test of treatment by LDL on acne vulgaris through gene HMGCR.


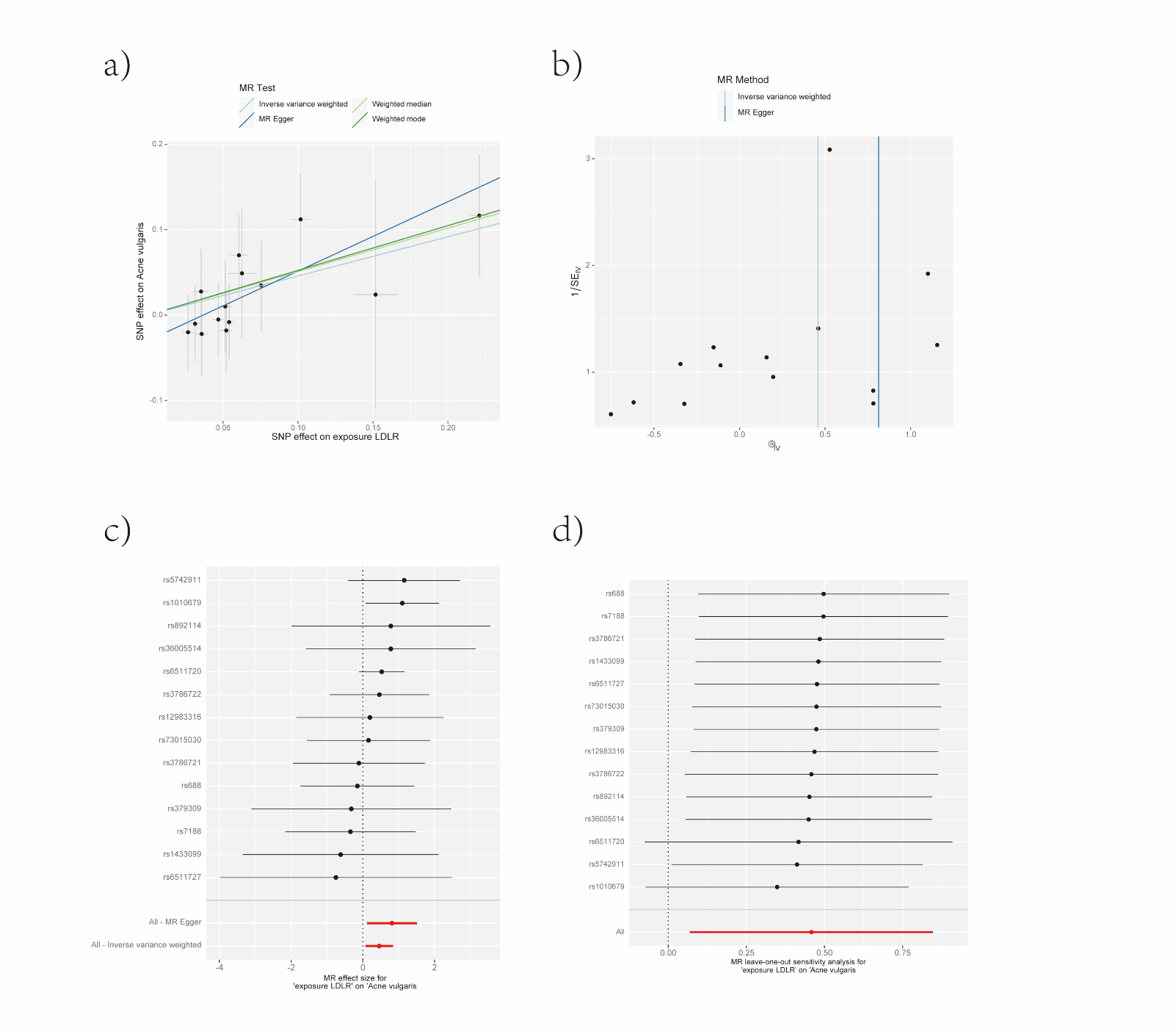


Figure S4. a) Scatter diagram b) funnel plot c) forest plot and d) leave-one-out test of treatment by LDL on acne vulgaris through gene LDLR.


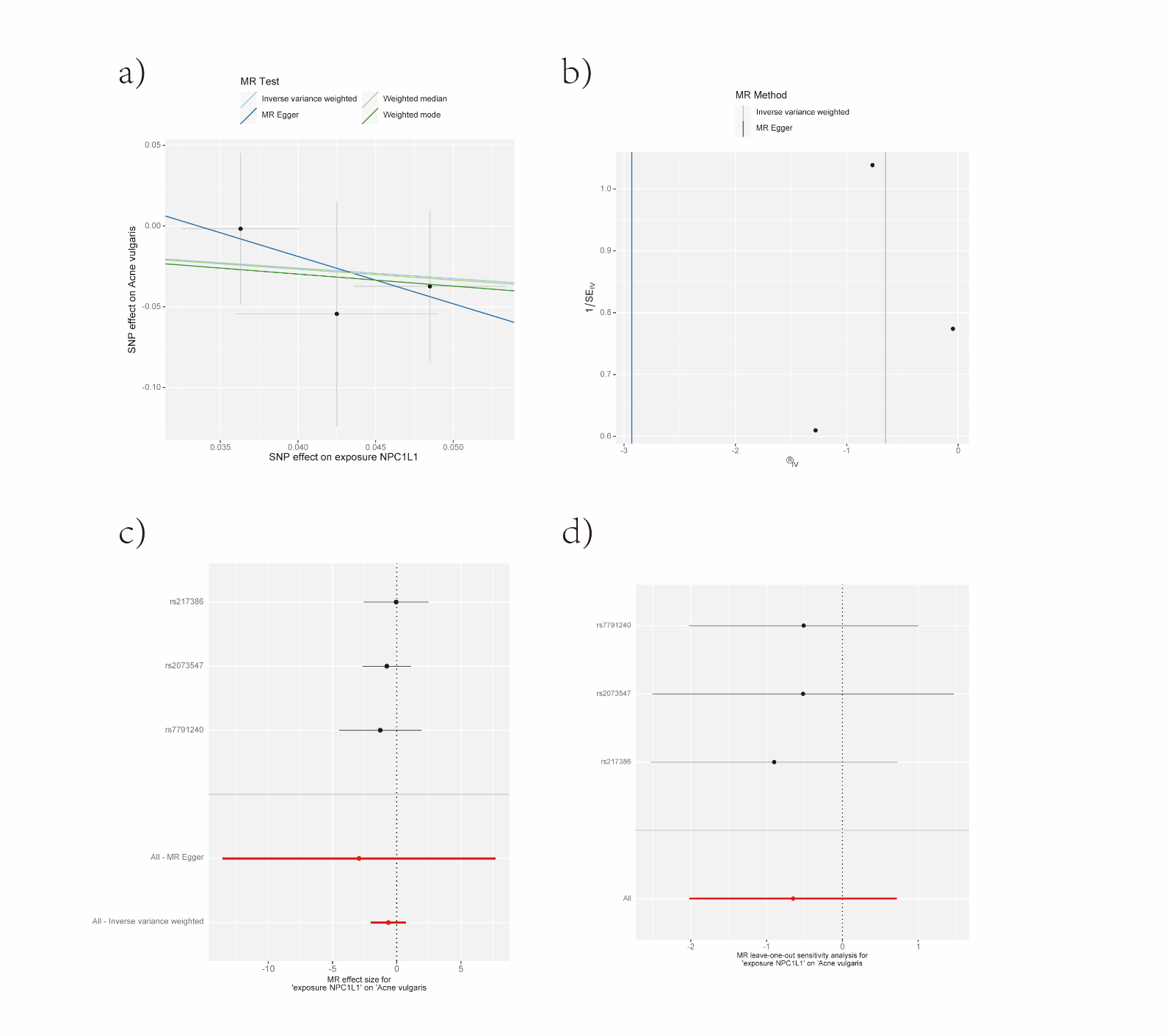


Figure S5. a) Scatter diagram b) funnel plot c) forest plot and d) leave-one-out test of treatment by LDL on acne vulgaris through gene NPC1L1.


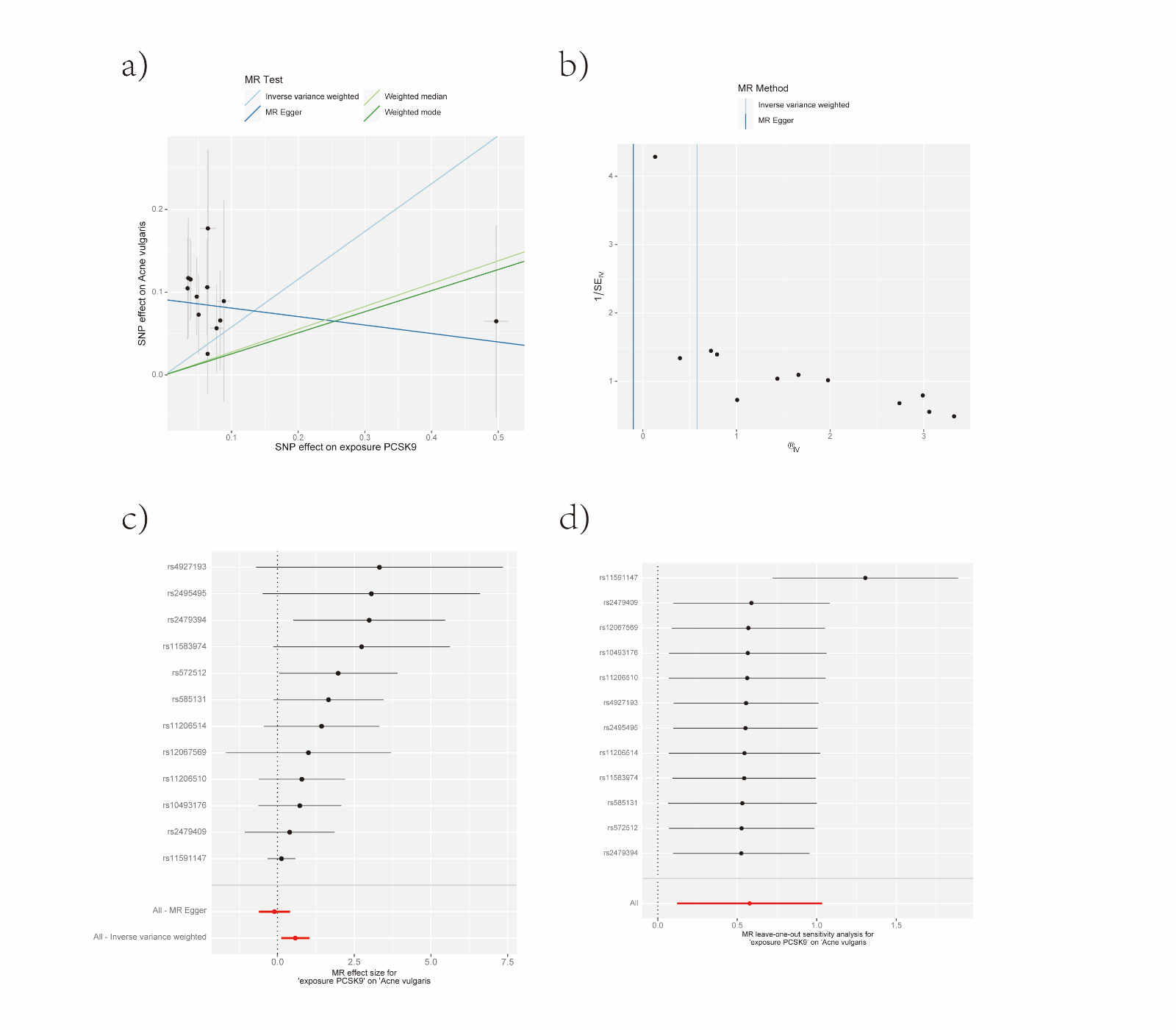


Figure S6. a) Scatter diagram b) funnel plot c) forest plot and d) leave-one-out test of treatment by LDL on acne vulgaris through gene PCSK9.


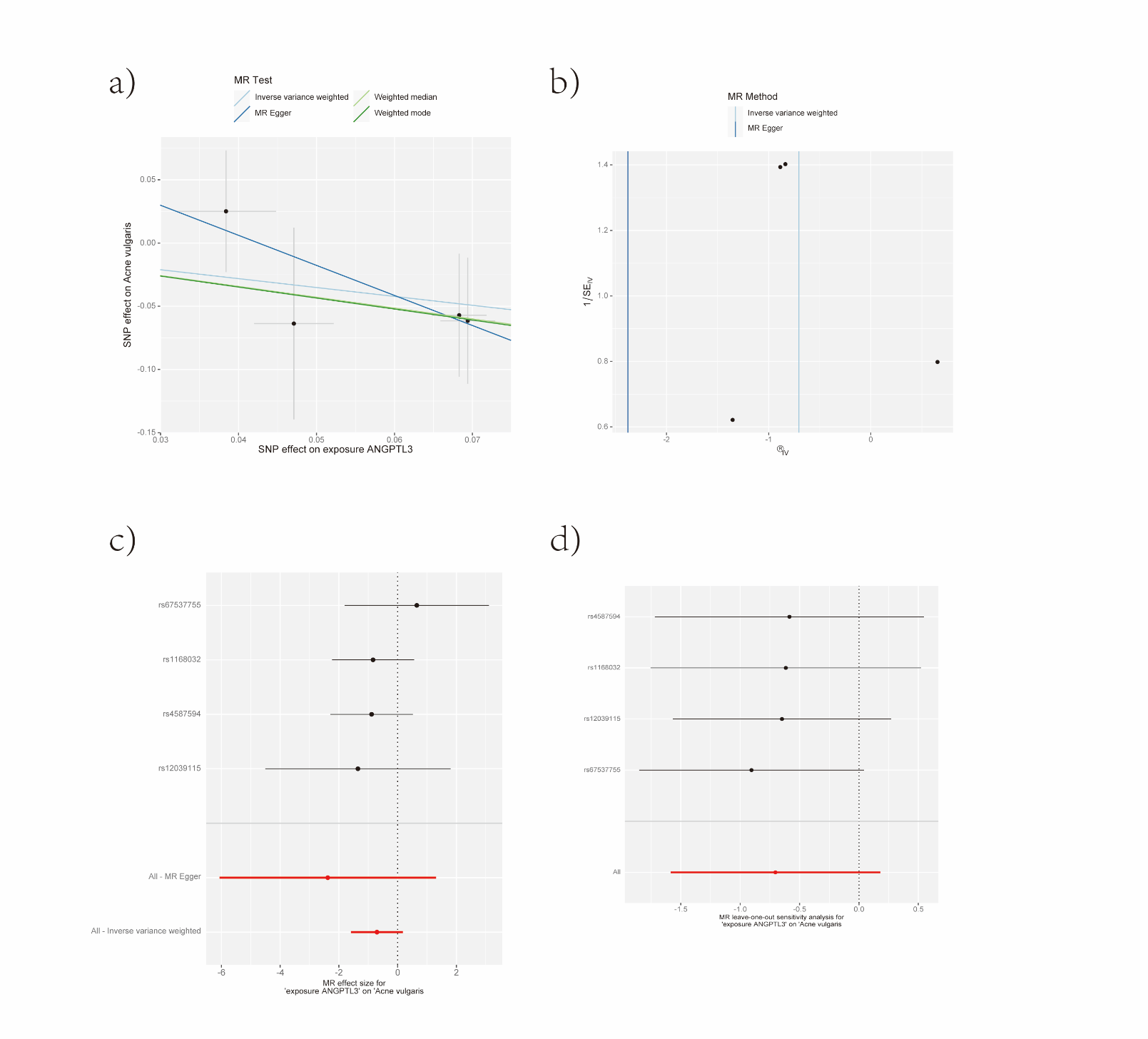


Figure S7. a) Scatter diagram b) funnel plot c) forest plot and d) leave-one-out test of treatment by TG on acne vulgaris through gene ANGPTL3.


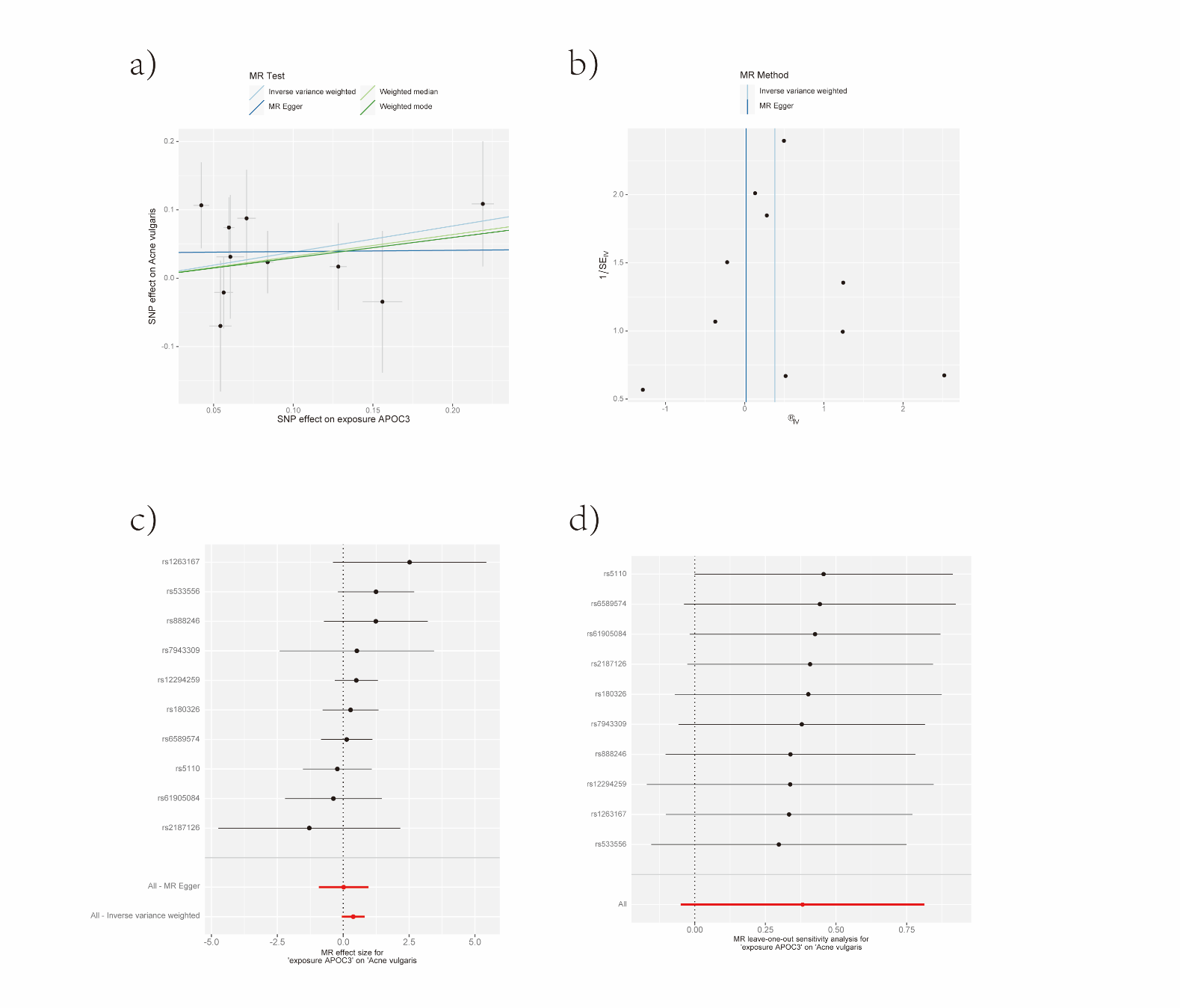


Figure S8. a) Scatter diagram b) funnel plot c) forest plot and d) leave-one-out test of treatment by TG on acne vulgaris through gene APOC3.


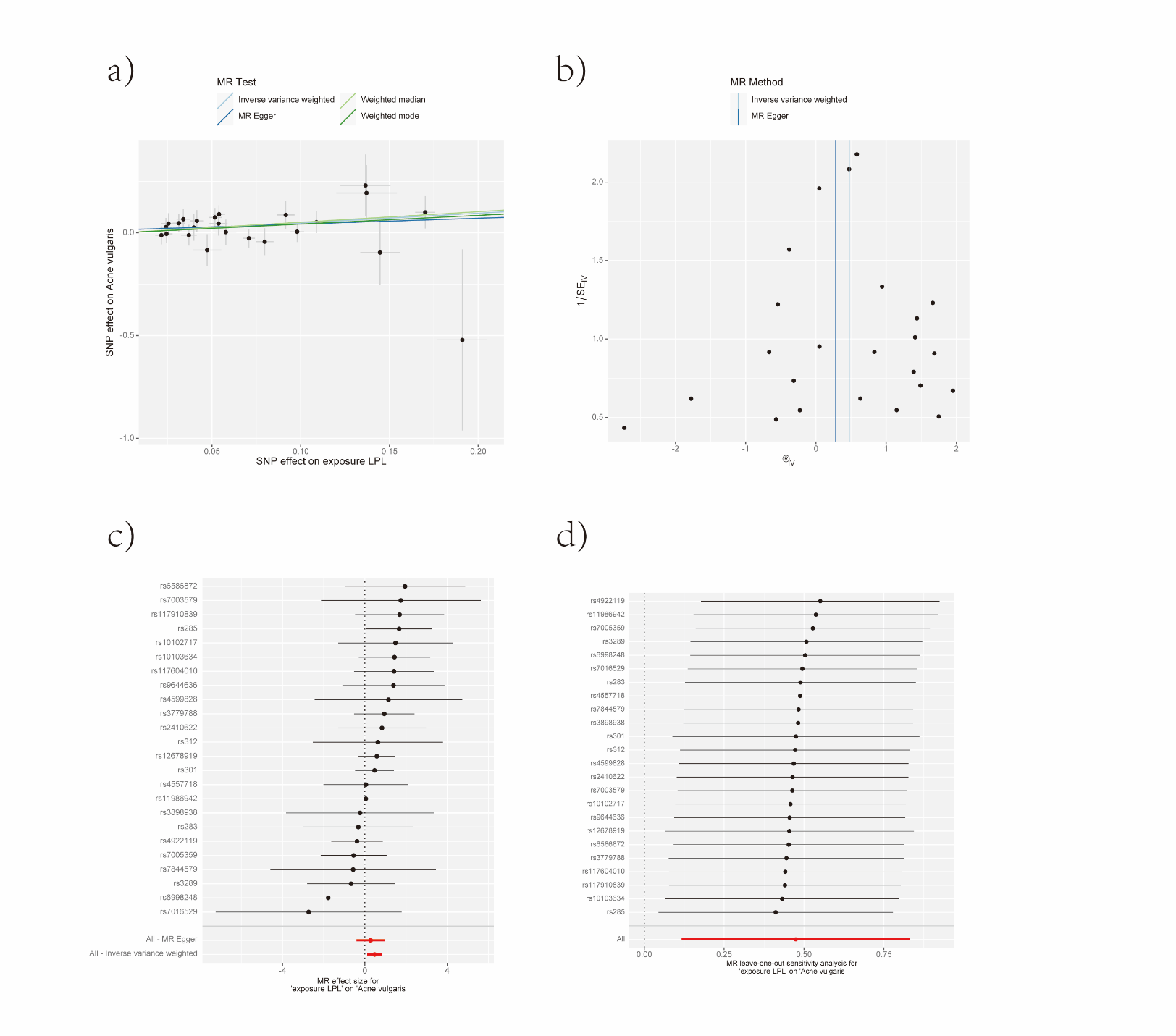


Figure S9. a) Scatter diagram b) funnel plot c) forest plot and d) leave-one-out test of treatment by TG on acne vulgaris through gene LPL.
