# Supplementary material for: Repurposing lipid-lowering drugs as potential treatment for acne vulgaris: a Mendelian randomization study
Source: Front Med (Lausanne). 2024 Jun 6;11:1385948. doi: 10.3389/fmed.2024.1385948 (PMC11187329; doi:10.3389/fmed.2024.1385948)
Supplement: Supplementary file 2 [file Data_Sheet_2.DOCX]

**Table S1.** Information of genetic instrumental variables used in the IVW-MR method to represent the exposure to statins therapy (lowering LDL-cholesterol) on acne vulgaris.

| **Exposure** | **SNP** | **EA** | **NEA** | **Chr** | **Beta** | **Se** | **Eaf** | **F-value** | **Pval** |
| --- | --- | --- | --- | --- | --- | --- | --- | --- | --- |
| Statins therapy | rs10066707 | A | G | 5 | 0.0497 | 0.0054 | 0.4169 | 108.0766 | 2.97E-19 |
| Statins therapy | rs10515198 | A | G | 5 | 0.0599 | 0.0061 | 0.1029 | 114.6832 | 5.99E-22 |
| Statins therapy | rs12659791 | C | T | 5 | 0.0433 | 0.005 | 0.1557 | 85.32802 | 1.42E-18 |
| Statins therapy | rs12916 | C | T | 5 | 0.0733 | 0.0038 | 0.4314 | 444.9357 | 7.79E-78 |
| Statins therapy | rs3804231 | A | G | 5 | 0.0642 | 0.0053 | 0.1319 | 163.4617 | 1.88E-29 |
| Statins therapy | rs3857388 | C | T | 5 | 0.0421 | 0.0059 | 0.128 | 68.45121 | 2.20E-11 |
| Statins therapy | rs72633962 | C | T | 5 | 0.06 | 0.0072 | 0.1412 | 72.61811 | 3.33E-15 |
|  |  |  |  |  |  |  |  |  |  |

The 7 SNPs (MAF>1%), located within ±100 kb windows from HMGCR gene, showed no significant association with acne vulgaris (p<5e^-8^). SNP: single nucleotide polymorphism; EA: effect allele; NEA, non-effect allele; EAF: effect allele frequency; Chr: chromosome; SE: standard error.

**Table S2.** Information of genetic instrumental variables used in the IVW-MR method to represent the exposure to Alirocumab therapy (lowering LDL-cholesterol) on acne vulgaris.

| **Exposure** | **SNP** | **EA** | **NEA** | **Chr** | **Beta** | **Se** | **Eaf** | **F-value** | **Pval** |
| --- | --- | --- | --- | --- | --- | --- | --- | --- | --- |
| Alirocumab therapy | rs10493176 | T | G | 1 | 0.0776 | 0.0102 | 0.8852 | 105.4483 | 2.54E-14 |
| Alirocumab therapy | rs11206510 | T | C | 1 | 0.0831 | 0.005 | 0.8456 | 312.1741 | 2.38E-53 |
| Alirocumab therapy | rs11206514 | A | C | 1 | 0.0507 | 0.0041 | 0.6108 | 211.68 | 9.95E-33 |
| Alirocumab therapy | rs11583974 | A | G | 1 | 0.0646 | 0.0117 | 0.03034 | 24.54903 | 3.95E-09 |
| Alirocumab therapy | rs11591147 | G | T | 1 | 0.497 | 0.018 | 0.98285 | 650.0564 | 8.58E-143 |
| Alirocumab therapy | rs12067569 | A | G | 1 | 0.0885 | 0.01 | 0.0343 | 85.27384 | 1.97E-17 |
| Alirocumab therapy | rs2479394 | G | A | 1 | 0.0386 | 0.0041 | 0.285 | 105.0854 | 1.58E-19 |
| Alirocumab therapy | rs2479409 | G | A | 1 | 0.0642 | 0.0041 | 0.3325 | 317.0327 | 2.52E-50 |
| Alirocumab therapy | rs2495495 | T | C | 1 | 0.0342 | 0.0059 | 0.1346 | 44.26404 | 3.52E-08 |
| Alirocumab therapy | rs4927193 | T | C | 1 | 0.0352 | 0.0056 | 0.8694 | 48.69271 | 4.27E-11 |
| Alirocumab therapy | rs572512 | T | C | 1 | 0.0478 | 0.0047 | 0.3456 | 155.7641 | 5.31E-26 |
| Alirocumab therapy | rs585131 | T | C | 1 | 0.0637 | 0.005 | 0.8153 | 205.2725 | 2.70E-35 |
|  |  |  |  |  |  |  |  |  |  |

The 12 SNPs (MAF>1%), located within ±100 kb windows from PCSK9 gene, showed powerful association with acne vulgaris (p<5e^-8^). SNP: single nucleotide polymorphism; EA: effect allele; NEA, non-effect allele; EAF: effect allele frequency; Chr: chromosome; SE: standard error.

**Table S3.** Information of genetic instrumental variables used in the IVW-MR method to represent the exposure to Ezetimibe therapy (lowering LDL-cholesterol) on acne vulgaris.

| **Exposure** | **SNP** | **EA** | **NEA** | **Chr** | **Beta** | **Se** | **Eaf** | **F-value** | **Pval** |
| --- | --- | --- | --- | --- | --- | --- | --- | --- | --- |
| Ezetimibe therapy | rs2073547 | G | A | 7 | 0.0485 | 0.0049 | 0.1939 | 125.0143 | 1.92E-21 |
| Ezetimibe therapy | rs217386 | G | A | 7 | 0.0363 | 0.0038 | 0.5923 | 110.1783 | 1.20E-19 |
| Ezetimibe therapy | rs7791240 | C | T | 7 | 0.0425 | 0.0065 | 0.09103 | 48.391 | 1.84E-10 |
|  |  |  |  |  |  |  |  |  |  |

The 3 SNPs (MAF>1%), located within ±100 kb windows from NPC1L1 gene, showed no significant association with acne vulgaris (p<5e^-8^). SNP: single nucleotide polymorphism; EA: effect allele; NEA, non-effect allele; EAF: effect allele frequency; Chr: chromosome; SE: standard error.

**Table S4.** Information of genetic instrumental variables used in the IVW-MR method to represent the exposure to Mipomersen therapy (lowering LDL-cholesterol) on acne vulgaris.

| **Exposure** | **SNP** | **EA** | **NEA** | **Chr** | **Beta** | **Se** | **Eaf** | **F-value** | **Pval** |
| --- | --- | --- | --- | --- | --- | --- | --- | --- | --- |
| Mipomersen therapy | rs10164442 | G | A | 2 | 0.0286 | 0.0039 | 0.3206 | 61.66756 | 0.0039 |
| Mipomersen therapy | rs10198175 | A | G | 2 | 0.0768 | 0.0063 | 0.08443 | 157.8775 | 0.0063 |
| Mipomersen therapy | rs12691202 | C | T | 2 | 0.0966 | 0.0114 | 0.94855 | 99.45455 | 0.0114 |
| Mipomersen therapy | rs12720796 | C | A | 2 | 0.0909 | 0.0141 | 0.02243 | 55.71849 | 0.0141 |
| Mipomersen therapy | rs12720842 | C | T | 2 | 0.0993 | 0.0116 | 0.02111 | 65.75929 | 0.0116 |
| Mipomersen therapy | rs13392272 | T | C | 2 | 0.0875 | 0.0037 | 0.4129 | 644.3717 | 0.0037 |
| Mipomersen therapy | rs1367117 | A | G | 2 | 0.1186 | 0.004 | 0.2876 | 1002.955 | 0.004 |
| Mipomersen therapy | rs17398765 | G | A | 2 | 0.0916 | 0.0076 | 0.0686 | 180.4382 | 0.0076 |
| Mipomersen therapy | rs1801701 | T | C | 2 | 0.0638 | 0.0064 | 0.07916 | 102.7391 | 0.0064 |
| Mipomersen therapy | rs3791981 | A | G | 2 | 0.0939 | 0.0067 | 0.8799 | 301.4884 | 0.0067 |
| Mipomersen therapy | rs4128553 | T | C | 2 | 0.0652 | 0.006 | 0.09894 | 131.2454 | 0.006 |
| Mipomersen therapy | rs4665788 | T | C | 2 | 0.0667 | 0.0042 | 0.2256 | 257.2097 | 0.0042 |
| Mipomersen therapy | rs515135 | C | T | 2 | 0.1394 | 0.0048 | 0.7823 | 1152.883 | 0.0048 |
| Mipomersen therapy | rs531593 | A | G | 2 | 0.0694 | 0.0078 | 0.93536 | 97.48214 | 0.0078 |
| Mipomersen therapy | rs6413458 | G | A | 2 | 0.094 | 0.0139 | 0.97625 | 65.19777 | 0.0139 |
| Mipomersen therapy | rs6547409 | C | T | 2 | 0.1226 | 0.0087 | 0.94063 | 280.9993 | 0.0087 |
| Mipomersen therapy | rs6725189 | G | T | 2 | 0.0604 | 0.0045 | 0.7652 | 227.0885 | 0.0045 |
| Mipomersen therapy | rs6754295 | T | G | 2 | 0.0628 | 0.0042 | 0.7414 | 262.0363 | 0.0042 |
| Mipomersen therapy | rs6756743 | T | C | 2 | 0.0553 | 0.0092 | 0.04222 | 40.53163 | 0.0092 |
| Mipomersen therapy | rs7567653 | G | A | 2 | 0.1145 | 0.0112 | 0.96306 | 150.4866 | 0.0112 |
|  |  |  |  |  |  |  |  |  |  |
|  |  |  |  |  |  |  |  |  |  |

The 20 SNPs (MAF>1%), located within ±100 kb windows from APOB gene, showed no significant association with acne vulgaris (p<5e^-8^). SNP: single nucleotide polymorphism; EA: effect allele; NEA, non-effect allele; EAF: effect allele frequency; Chr: chromosome; SE: standard error.

**Table S5.** Information of genetic instrumental variables used in the IVW-MR method to represent the exposure to Evinacumab therapy (lowering LDL-cholesterol) on acne vulgaris.

| **Exposure** | **SNP** | **EA** | **NEA** | **Chr** | **Beta** | **Se** | **Eaf** | **F-value** | **Pval** |
| --- | --- | --- | --- | --- | --- | --- | --- | --- | --- |
| Evinacumab therapy | rs11485618 | A | G | 1 | 0.05 | 0.0039 | 0.6913 | 181.6526 | 3.73E-33 |
| Evinacumab therapy | rs12039115 | C | T | 1 | 0.0345 | 0.0057 | 0.8905 | 40.16573 | 1.26E-09 |
| Evinacumab therapy | rs1627591 | T | C | 1 | 0.049 | 0.0039 | 0.69 | 177.8845 | 1.46E-32 |
|  |  |  |  |  |  |  |  |  |  |

The 3 SNPs (MAF>1%), located within ±100 kb windows from ANGPTL3 gene, showed no significant association with acne vulgaris (p<5e^-8^). SNP: single nucleotide polymorphism; EA: effect allele; NEA, non-effect allele; EAF: effect allele frequency; Chr: chromosome; SE: standard error.

**Table S6.** Information of genetic instrumental variables used in the IVW-MR method to represent the exposure to Fenofibrate therapy (lowering LDL-cholesterol) on acne vulgaris.

| **Exposure** | **SNP** | **EA** | **NEA** | **Chr** | **Beta** | **Se** | **Eaf** | **F-value** | **Pval** |
| --- | --- | --- | --- | --- | --- | --- | --- | --- | --- |
| Fenofibrate therapy | rs4253776 | G | A | 2 | 0.0311 | 0.0059 | 0.124 | 35.95331 | 3.35E-08 |
|  |  |  |  |  |  |  |  |  |  |

The 1 SNPs (MAF>1%), located within ±100 kb windows from PPARA gene, showed no significant association with acne vulgaris (p<5e^-8^). SNP: single nucleotide polymorphism; EA: effect allele; NEA, non-effect allele; EAF: effect allele frequency; Chr: chromosome; SE: standard error.

**Table S7.** Information of genetic instrumental variables used in the IVW-MR method to represent the exposure to LDLR (lowering LDL-cholesterol) on acne vulgaris.

| **Exposure** | **SNP** | **EA** | **NEA** | **Chr** | **Beta** | **Se** | **Eaf** | **F-value** | **Pval** |
| --- | --- | --- | --- | --- | --- | --- | --- | --- | --- |
| Acipimox therapy | rs1010679 | T | C | 19 | 0.1017 | 0.0063 | 0.8206 | 253.6548 | 3.51E-54 |
| Acipimox therapy | rs12983316 | G | A | 19 | 0.0514 | 0.0052 | 0.1689 | 122.1983 | 7.44E-22 |
| Acipimox therapy | rs1433099 | C | T | 19 | 0.0357 | 0.0043 | 0.7098 | 90.0785 | 2.51E-16 |
| Acipimox therapy | rs36005514 | A | G | 19 | 0.0626 | 0.0096 | 0.0752 | 45.28756 | 7.06E-10 |
| Acipimox therapy | rs3786721 | T | C | 19 | 0.0468 | 0.0038 | 0.4617 | 178.3078 | 2.89E-31 |
| Acipimox therapy | rs3786722 | C | A | 19 | 0.0754 | 0.0043 | 0.7573 | 358.8064 | 5.52E-63 |
| Acipimox therapy | rs379309 | C | T | 19 | 0.0313 | 0.0039 | 0.5026 | 81.75329 | 1.39E-13 |
| Acipimox therapy | rs5742911 | A | G | 19 | 0.0606 | 0.0057 | 0.7322 | 111.877 | 4.83E-24 |
| Acipimox therapy | rs6511720 | G | T | 19 | 0.2209 | 0.0061 | 0.90237 | 1479.563 | 1.00E-200 |
| Acipimox therapy | rs6511727 | T | G | 19 | 0.0266 | 0.0038 | 0.3852 | 57.07067 | 1.84E-11 |
| Acipimox therapy | rs688 | T | C | 19 | 0.054 | 0.0037 | 0.4472 | 240.8154 | 1.01E-43 |
| Acipimox therapy | rs7188 | C | A | 19 | 0.0521 | 0.0043 | 0.3259 | 172.6098 | 9.39E-31 |
| Acipimox therapy | rs73015030 | G | A | 19 | 0.1517 | 0.0148 | 0.97493 | 93.52888 | 2.62E-22 |
| Acipimox therapy | rs892114 | A | G | 19 | 0.0353 | 0.0047 | 0.2269 | 68.36379 | 7.63E-13 |
|  |  |  |  |  |  |  |  |  |  |

The 14 SNPs (MAF>1%), located within ±100 kb windows from LDLR gene, showed powerful association with acne vulgaris (p<5e^-8^). SNP: single nucleotide polymorphism; EA: effect allele; NEA, non-effect allele; EAF: effect allele frequency; Chr: chromosome; SE: standard error.

**Table S8.** Information of genetic instrumental variables used in the IVW-MR method to represent the exposure to Evinacumab therapy (lowering triglyceride) on acne vulgaris.

| **Exposure** | **SNP** | **EA** | **NEA** | **Chr** | **Beta** | **Se** | **Eaf** | **F-value** | **Pval** |
| --- | --- | --- | --- | --- | --- | --- | --- | --- | --- |
| Evinacumab therapy | rs1168032 | G | A | 1 | 0.0683 | 0.0035 | 0.6781 | 362.8053 | 1.49E-80 |
| Evinacumab therapy | rs12039115 | C | T | 1 | 0.0471 | 0.0051 | 0.8905 | 76.93946 | 6.54E-21 |
| Evinacumab therapy | rs4587594 | G | A | 1 | 0.0694 | 0.0035 | 0.69 | 367.0405 | 3.50E-82 |
| Evinacumab therapy | rs67537755 | A | G | 1 | 0.0384 | 0.0064 | 0.2573 | 31.15579 | 5.96E-09 |
|  |  |  |  |  |  |  |  |  |  |

The 4 SNPs (MAF>1%), located within ±100 kb windows from ANGPTL3 gene, showed no significant association with acne vulgaris (p<5e^-8^). SNP: single nucleotide polymorphism; EA: effect allele; NEA, non-effect allele; EAF: effect allele frequency; Chr: chromosome; SE: standard error.

**Table S9.** Information of genetic instrumental variables used in the IVW-MR method to represent the exposure to Volanesorsen therapy (lowering triglyceride) on acne vulgaris.

| **Exposure** | **SNP** | **EA** | **NEA** | **Chr** | **Beta** | **Se** | **Eaf** | **F-value** | **Pval** |
| --- | --- | --- | --- | --- | --- | --- | --- | --- | --- |
| Volanesorsen therapy | rs12294259 | T | C | 11 | 0.219 | 0.0069 | 0.05937 | 927.6235 | 1.79E-200 |
| Volanesorsen therapy | rs1263167 | A | G | 11 | 0.0423 | 0.0049 | 0.8193 | 77.75012 | 5.59E-17 |
| Volanesorsen therapy | rs180326 | G | T | 11 | 0.0839 | 0.0036 | 0.3628 | 527.846 | 6.27E-108 |
| Volanesorsen therapy | rs2187126 | A | G | 11 | 0.0543 | 0.0069 | 0.94591 | 53.65245 | 2.90E-15 |
| Volanesorsen therapy | rs5110 | A | C | 11 | 0.156 | 0.0124 | 0.06464 | 241.1887 | 2.14E-34 |
| Volanesorsen therapy | rs533556 | A | C | 11 | 0.0596 | 0.0035 | 0.3694 | 291.5389 | 1.61E-59 |
| Volanesorsen therapy | rs61905084 | T | C | 11 | 0.0563 | 0.0059 | 0.8351 | 73.61748 | 2.55E-20 |
| Volanesorsen therapy | rs6589574 | A | G | 11 | 0.1283 | 0.0054 | 0.1214 | 626.4542 | 3.03E-113 |
| Volanesorsen therapy | rs7943309 | G | A | 11 | 0.0605 | 0.0087 | 0.96042 | 48.07714 | 1.16E-11 |
| Volanesorsen therapy | rs888246 | T | C | 11 | 0.0707 | 0.0058 | 0.08179 | 119.3077 | 8.99E-31 |
|  |  |  |  |  |  |  |  |  |  |

The 10 SNPs (MAF>1%), located within ±100 kb windows from APOC3 gene, showed no significant association with acne vulgaris (p<5e^-8^). SNP: single nucleotide polymorphism; EA: effect allele; NEA, non-effect allele; EAF: effect allele frequency; Chr: chromosome; SE: standard error.

**Table S10.** Information of genetic instrumental variables used in the IVW-MR method to represent the exposure to LPL (lowering triglyceride) on acne vulgaris.

| **Exposure** | **SNP** | **EA** | **NEA** | **Chr** | **Beta** | **Se** | **Eaf** | **F-value** | **Pval** |
| --- | --- | --- | --- | --- | --- | --- | --- | --- | --- |
| Acipimox therapy | rs10102717 | C | T | 8 | 0.0313 | 0.0034 | 0.591 | 84.21768 | 9.66E-20 |
| Acipimox therapy | rs10103634 | G | A | 8 | 0.0517 | 0.0036 | 0.6702 | 194.0227 | 8.09E-46 |
| Acipimox therapy | rs117604010 | G | A | 8 | 0.1371 | 0.017 | 0.98021 | 59.27298 | 3.11E-15 |
| Acipimox therapy | rs117910839 | T | A | 8 | 0.1365 | 0.0141 | 0.96306 | 115.1203 | 7.07E-20 |
| Acipimox therapy | rs11986942 | C | G | 8 | 0.098 | 0.0038 | 0.6755 | 712.4413 | 3.17E-142 |
| Acipimox therapy | rs12678919 | A | G | 8 | 0.1702 | 0.0056 | 0.8786 | 1105.237 | 1.82E-199 |
| Acipimox therapy | rs2410622 | T | C | 8 | 0.0537 | 0.0051 | 0.1438 | 122.5553 | 1.20E-26 |
| Acipimox therapy | rs283 | T | C | 8 | 0.037 | 0.0044 | 0.2348 | 83.60296 | 1.70E-16 |
| Acipimox therapy | rs285 | C | T | 8 | 0.054 | 0.0034 | 0.4723 | 251.8172 | 4.80E-56 |
| Acipimox therapy | rs301 | T | C | 8 | 0.1089 | 0.0039 | 0.5726 | 1037.726 | 1.86E-167 |
| Acipimox therapy | rs312 | G | C | 8 | 0.0398 | 0.005 | 0.8799 | 59.50208 | 4.93E-17 |
| Acipimox therapy | rs3289 | C | T | 8 | 0.1447 | 0.0111 | 0.0277 | 191.3872 | 3.67E-33 |
| Acipimox therapy | rs3779788 | C | T | 8 | 0.0916 | 0.0049 | 0.8694 | 339.1597 | 4.66E-76 |
| Acipimox therapy | rs3898938 | C | T | 8 | 0.0244 | 0.0034 | 0.5607 | 52.10555 | 1.22E-12 |
| Acipimox therapy | rs4557718 | C | T | 8 | 0.0578 | 0.0059 | 0.1266 | 126.1322 | 3.26E-24 |
| Acipimox therapy | rs4599828 | C | T | 8 | 0.0241 | 0.0034 | 0.533 | 51.40979 | 1.59E-11 |
| Acipimox therapy | rs4922119 | C | T | 8 | 0.0708 | 0.0034 | 0.5501 | 428.5345 | 1.67E-95 |
| Acipimox therapy | rs6586872 | G | A | 8 | 0.0339 | 0.0038 | 0.7441 | 77.81744 | 4.09E-19 |
| Acipimox therapy | rs6998248 | T | C | 8 | 0.0473 | 0.0079 | 0.8681 | 46.6534 | 6.75E-10 |
| Acipimox therapy | rs7003579 | T | C | 8 | 0.0255 | 0.004 | 0.215 | 39.01535 | 4.72E-10 |
| Acipimox therapy | rs7005359 | A | G | 8 | 0.0797 | 0.005 | 0.7942 | 294.55 | 1.86E-57 |
| Acipimox therapy | rs7016529 | C | T | 8 | 0.1911 | 0.014 | 0.01319 | 146.5548 | 3.57E-35 |
| Acipimox therapy | rs7844579 | C | T | 8 | 0.0215 | 0.0034 | 0.5264 | 40.26319 | 3.00E-10 |
| Acipimox therapy | rs9644636 | G | T | 8 | 0.0415 | 0.0039 | 0.2639 | 118.8935 | 2.02E-27 |
|  |  |  |  |  |  |  |  |  |  |

The 24 SNPs (MAF>1%), located within ±100 kb windows from LPL gene, showed powerful association with acne vulgaris (p<5e^-8^). SNP: single nucleotide polymorphism; EA: effect allele; NEA, non-effect allele; EAF: effect allele frequency; Chr: chromosome; SE: standard error.

**Table S11.** Sensitivity analysis of the association analysis between different lowering LDL-cholesterol therapy and coronary heart disease through MR Egger and inverse variance weighted (IVW) method.

|  | **Data source** | **MR Egger method** |  | **IVW method** |  | **I^2^ test** |
| --- | --- | --- | --- | --- | --- | --- |
| Targets |  | Q value | p | Q value | p | I^2^ value |
| HMGCR | GLGC | 3.162 | 0.675 | 4.685 | 0.585 | 0 |
| PCSK9 | GLGC | 9.383 | 0.311 | 9.773 | 0.369 | 7.905 |
| NPC1L1 | GLGC | 0.002 | 0.967 | 0.209 | 0.901 | 0 |
| APOB | GLGC | 22.267 | 0.175 | 31.820 | 0.023 | 43.432 |
| ANGPTL3 | GLGC | 0.008 | 0.931 | 0.060 | 0.970 | 0 |
| LDLR | GLGC | 5.185 | 0.738 | 5.196 | 0.817 | 0 |

**Table S12.** Sensitivity analysis of the association analysis between different lowering triglyceride therapy and coronary heart disease through MR Egger and inverse variance weighted (IVW) method.

|  | **Data source** | **MR Egger method** |  | **IVW method** |  | **I^2^ test** |
| --- | --- | --- | --- | --- | --- | --- |
| Targets |  | Q value | p | Q value | p | I^2^ value |
| ANGPTL3 | GLGC | 0.369 | 0.831 | 0.693 | 0.875 | 0 |
| APOC3 | GLGC | 11.691 | 0.166 | 11.957 | 0.216 | 24.732 |
| LPL | GLGC | 16.128 | 0.709 | 24.937 | 0.250 | 15.789 |

**Table S13.** Sensitivity analysis of the association analysis between different lowering LDL-cholesterol therapy and acne vulgaris through MR Egger and inverse variance weighted (IVW) method.

|  | **Data source** | **MR Egger method** |  | **IVW method** |  | **I^2^ test** |
| --- | --- | --- | --- | --- | --- | --- |
| Targets |  | Q value | p | Q value | p | I^2^ value |
| HMGCR | GLGC | 3.221 | 0.666 | 3.235 | 0.779 | 0 |
| PCSK9 | GLGC | 3.654 | 0.962 | 17.660 | 0.090 | 37.713 |
| NPC1L1 | GLGC | 0.201 | 0.654 | 0.381 | 0.827 | 0 |
| APOB | GLGC | 31.261 | 0.027 | 31.460 | 0.036 | 39.606 |
| LDLR | GLGC | 4.328 | 0.977 | 5.757 | 0.955 | 0 |

**Table S14.** Sensitivity analysis of the association analysis between different lowering triglyceride therapy and acne vulgaris through MR Egger and inverse variance weighted (IVW) method.

|  | **Data source** | **MR Egger method** |  | **IVW method** |  | **I^2^ test** |
| --- | --- | --- | --- | --- | --- | --- |
| Targets |  | Q value | p | Q value | p | I^2^ value |
| ANGPTL3 | GLGC | 0.596 | 0.742 | 1.436 | 0.697 | 0 |
| LPL | GLGC | 18.077 | 0.701 | 18.495 | 0.730 | 0 |
